# Study on the operational efficiency of clinical departments in a Grade III general hospital

**DOI:** 10.3389/fpubh.2026.1965710

**Published:** 2026-09-29

**Authors:** Ye Nie, LiangRong Zhou, Chao Huang, Ying Du

**Affiliations:** College of Humanities and Management, Hunan University of Chinese Medicine, Changsha, China

**Keywords:** clinical departments, data envelopment analysis, hospital management, operational efficiency, resource allocation

## Abstract

**Objective:**

To analyze the operational efficiency of clinical departments in tertiary general hospitals, thereby providing a basis for optimizing management processes and adjusting the allocation of departmental resources.

**Methods:**

Given the variations in the size of clinical departments and the difficulty of rapidly adjusting resource allocation in the short term, this study employed the DEA-BCC model to analyze efficiency. Using the DEA composite technical efficiency score as the dependent variable and incorporating three environmental variables—departmental characteristics, designation as a national or provincial key specialty, and the proportion of doctors holding senior professional titles—a Tobit regression model was utilized to analyze the factors influencing the operational efficiency of clinical departments.

**Results:**

The comprehensive technical efficiency of this clinical department was 0.756, the pure technical efficiency was 0.856, and the scale efficiency was 0.888. Tobit analysis revealed a significant positive correlation between the proportion of senior-level physicians and the comprehensive technical efficiency of clinical departments; neither the department’s attributes nor its status as a national or provincial key speciality reached statistical significance.

**Discussion:**

There are disparities in operational efficiency across clinical departments, with some departments exhibiting a mismatch between inputs and outputs. It is necessary to optimize resource allocation in line with each department’s functional role and the characteristics of returns to scale while enhancing the capacity to diagnose and treat complex and critical cases, thereby achieving a synergistic improvement in both efficiency and healthcare service capacity. Compared with previous efficiency studies that treated the hospital as a whole as the decision-making unit, this study focuses on clinical departments within a tertiary general hospital. It utilizes the CMI to adjust for differences in case mix and combines this with DEA efficiency analysis, slack variables, sensitivity analysis, and department-specific contextual variables to identify variations in resource allocation at the departmental level, thereby providing guidance for refined resource management in hospitals.

## 1 Introduction

The 2026 National Health and Healthcare Work Conference proposed the principle of “strengthening the foundation, stabilizing the second tier, and controlling the third tier,” further clarifying the service boundaries of different healthcare institutions (1). According to the Evaluation Criteria for Tertiary Hospitals (2025 Edition), tertiary hospitals are mainly involved in “diagnosis and treatment of intractable and complex diseases” and “treatment of acute, critical, and severe conditions.” However, due to the overall resource constraints and the complexity of internal structures, some public hospitals are still facing challenges such as uneven resource distribution, lagging operational efficiency, and lack of incentives for innovation (2). In a social context where patients tend to seek treatment at tertiary hospitals, some of these institutions experience a “mismatch between roles and resources” concerning their functional positioning. This situation leads to high-quality medical resources being predominantly allocated to the treatment of common and prevalent diseases, which in turn hinders the hospitals’ ability to fully realize their capacity for treating acute, critical, and severe conditions, as well as complex and intractable diseases. This is a key factor limiting improvements in the operational efficiency of clinical departments (3, 4). As the core units responsible for delivering clinical care, the operational efficiency of clinical departments reflects, to a certain extent, the level of resource utilization and management effectiveness within a hospital (5). This study employs data envelopment analysis to assess the operational efficiency and resource distribution among different departments at a tertiary general hospital in Hunan Province. As a representative tertiary general hospital in the region, conducting an in-depth analysis of the operational efficiency of its clinical departments is crucial for addressing policies such as the “Tertiary Hospital Control” initiative and for enhancing the hospital’s overall operational efficiency.

## 2 Materials and methods

### 2.1 Data sources

The study focused on 23 clinical departments at a tertiary general hospital in Hunan Province. The data were sourced from medical record statistics, clinical operations, and relevant hospital management platforms across the various departments of the participating hospital. The data were collected between January and December 2025. The study utilized annual aggregated data at the departmental level; no personally identifiable information, such as patients’ names or identity card numbers, was included, and no clinical interventions were carried out.

### 2.2 Research methodology

This study employs a two-stage analytical approach. In the first stage, the DEA-BCC model is used to evaluate the relative technical efficiency of clinical departments; in the second stage, the Tobit model is used to analyze the statistical relationship between departmental contextual variables and DEA efficiency scores.

Data Envelopment Analysis (DEA) is an objective, non-parametric estimation method that evaluates the relative efficiency of comparable units of the same type based on multiple input and output indicators (6). Given that this study focuses on whether there is any redundancy in the allocation of resources such as beds and healthcare staff and whether resource inputs can be further reduced whilst maintaining a given level of service output, an input-oriented BCC model has been adopted. The BCC model assumes variable returns to scale, with aggregate technical efficiency derived as the product of pure technical efficiency and scale efficiency, thereby providing a more detailed measure of the decision-making unit’s aggregate efficiency (7). For departments that did not meet DEA efficiency, a further analysis of slack variables was conducted. Target values for each input and output were obtained based on the DEA optimal solution, and the slack was standardized into relative indicators. Specifically, the input redundancy rate is calculated as (actual input value—target input value)/actual input value; the output shortfall rate is calculated as (target output value—actual output value)/actual output value. An indicator value of 0 indicates that there is no slack in the corresponding dimension; a higher input redundancy ratio indicates a higher proportion of inputs that can be reduced, while a higher output deficiency ratio indicates a more pronounced output shortfall relative to the DEA projection target under current input conditions.

In the second stage, the comprehensive technical efficiency value calculated using DEA was taken as the dependent variable, while only department-specific contextual variables—including department attributes, designation as a national or provincial key, the proportion of doctors with senior professional titles, and the number of other healthcare professionals—were included as explanatory variables. DEA input–output indicators, such as the number of beds and the number of doctors, were no longer used directly as explanatory variables in the second stage in order to reduce the risk of spurious correlations and circular reasoning. As DEA efficiency scores range from 0 to 1, and the efficiency scores of some decision-making units are equal to 1, while the Tobit model is suitable for situations where the dependent variable has value boundaries—which can, to some extent, reduce the bias and inconsistency that may arise from ordinary least squares estimation—the Tobit model was therefore adopted for the analysis; however, its results should not be interpreted as causal effects (8). Previous studies on hospital efficiency have utilized the DEA-Tobit framework; however, as DEA efficiency scores are first-stage estimators and traditional second-stage Tobit models have limitations in statistical inference, the results of this study are interpreted solely as exploratory associations and have not been validated using methods such as bootstrap truncated regression.

### 2.3 Selection of evaluation indicators

This study selected the number of beds, the number of doctors, and the number of nurses as input indicators, while the output indicators included the number of outpatient visits, the number of CMI-adjusted discharged cases, and the reciprocal of the average length of stay, all based on a review of the relevant literature and consideration of the public welfare nature of public hospitals, as well as the representativeness and scientific rigor of the selected indicators (9). The formula for calculating the CMI-adjusted number of discharged cases is CMI-adjusted number of discharged cases = actual number of discharged cases in the department × the department’s annual CMI value. Previous studies have used the Case Mix Index to adjust the volume of inpatient services to mitigate the impact of differences in the complexity of patients’ conditions across different institutions on the comparison of service outputs (10).

When the CMI is 1.0, it indicates that the case mix complexity is equivalent to one standard case; the CMI-adjusted number of discharged cases is, in essence, the equivalent number of standard cases converted according to case mix complexity. To examine the impact of case mix adjustment on efficiency measurement results, a sensitivity analysis was conducted by substituting the actual number of discharged cases for the CMI-adjusted number of discharged cases while keeping other input–output indicators and model settings unchanged. The reciprocal of the average length of stay is primarily used as a proxy indicator of the efficiency of hospital resource utilization. As DEA output indicators are typically treated according to the principle that “a higher value indicates higher output,” this study has converted the average length of stay into its reciprocal, such that a shorter length of stay corresponds to a higher indicator value. It should be noted that a shorter average length of stay does not necessarily indicate higher quality of care or better patient outcomes. For tertiary hospitals treating complex and critically ill patients, the duration of hospitalization is influenced by factors such as the severity of the condition and the complexity of diagnosis and treatment (11). Therefore, this study interprets the reciprocal of the average length of stay solely as a proxy indicator of the efficiency of hospital resource turnover and does not equate it with the quality of care or the overall performance of the department.

## 3 Results

### 3.1 Descriptive analysis of input and output indicators

Among the 23 chosen clinical departments, 13 were categorized as medical and 10 as surgical. The divisions showed significant differences in their inputs and outputs. The average values for the input indicators were as follows: The total number of beds was 97.09, the number of doctors was 25.57, and the number of nurses was 48. The average values of the output metrics were 39,813 outpatient visits, 4,371.22 discharges, a CMI of 1.29, 5,274.88 CMI-adjusted discharges, and the inverse of the mean length of stay of 0.132. Further details are provided in Table 1.

**Table 1:** Overview of input and output in clinical departments.

Variable		Minimum	Maximum	Mean	Standard deviation
Inputs	Number of beds	30	211	97.09	62.2
	Number of doctors	8	72	25.57	16.49
	Number of nurses	15	130	48	31.8
Outputs	Number of outpatient visits	2,699	106,022	39,813	25992.79
	Number of discharges	574	11,459	4371.22	2947.67
	CMI score	0.65	3.42	1.29	0.71
	CMI-adjusted number of discharges	665.15	18759.53	5274.88	4439.60
	Inverse of average length of stay	0.05	0.27	0.132	0.049

### 3.2 Analysis of operational efficiency in hospital clinical departments

#### 3.2.1 DEA efficiency analysis of clinical departments

The average comprehensive technical efficiency across all clinical departments in the hospital was 0.756, with an average pure technical efficiency of 0.856 and an average scale efficiency of 0.888. In terms of department divisions, the total efficiency of the internal medicine system ranged from 0.447 to 1.000, with significant differences in efficiency among departments. The Oncology and Endocrinology departments were the only ones classified as DEA-efficient. Four departments, including cardiology and pediatrics, achieved a technical efficiency rank of 1; however, in view of scale efficiency constraints, there is room for optimization. The average comprehensive efficiency of the surgical departments was 0.82, which was generally better than that of the medical departments. Five departments, including Cardiothoracic Surgery and Ophthalmology, were classified as DEA-efficient. Departments such as Orthopaedic and Plastic Surgery, as well as Wound Care and Burns, have relatively low comprehensive efficiency and require further optimization of their scale or technical allocation.

With regard to returns to scale, only two departments in the internal medicine division exhibit constant returns to scale; the majority of departments have yet to achieve an optimal allocation of resources in beds and staff. In contrast, the scale efficiency of the surgical departments is close to 1, indicating a relatively reasonable allocation of resources, although a few departments still have potential for expansion. Refer to Table 2.

**Table 2:** Analysis of clinical department operational efficiency based on the DEA model.

Department category	Department name	Pure technical efficiency	Scale efficiency	Comprehensive technical efficiency	Effectiveness	Returns to scale
Internal Medicine	Cardiology	1	0.94	0.94	Weakly effective	drs
	Respiratory and critical care medicine	0.608	0.988	0.601	Inefficient	drs
	Integrative medicine	0.675	0.83	0.56	Inefficient	irs
	Gastroenterology	0.762	1	0.762	Inefficient	irs
	Neurology	0.52	0.984	0.512	Inefficient	drs
	Paediatrics	1	0.559	0.559	Weakly effective	drs
	Haematology	0.817	0.98	0.8	Inefficient	irs
	Oncology	1	1	1	Efficient	—
	Nephrology	0.559	0.897	0.501	Inefficient	irs
	Endocrinology	1	1	1	Efficient	—
	Infectious diseases	0.661	0.981	0.648	Inefficient	irs
	General practice	0.996	0.868	0.865	Inefficient	irs
	Rehabilitation medicine	0.745	0.601	0.447	Inefficient	irs
Surgery	Obstetrics and gynaecology	1	0.62	0.62	Weakly effective	drs
	Otolaryngology–head and neck surgery	1	1	1	Efficient	—
	Ophthalmology	1	1	1	Efficient	—
	Oral and maxillofacial surgery	1	1	1	Efficient	—
	Cardiothoracic surgery	1	1	1	Efficient	—
	Department of plastic surgery, wounds and burns	1	0.39	0.39	Weakly effective	irs
	Orthopaedics	0.801	0.839	0.672	Inefficient	drs
	General surgery	1	1	1	Efficient	—
	Neurosurgery	0.596	0.996	0.594	Inefficient	drs
	Urology	0.956	0.953	0.911	Inefficient	irs
Total	Average	0.856	0.888	0.756		

*irs, drs and—denote increasing returns to scale, decreasing returns to scale and constant returns to scale, respectively.

#### 3.2.2 Sensitivity analysis of clinical departments

Based on the sensitivity-modelled DEA analysis, the mean comprehensive technical efficiency was 0.709, the mean pure technical efficiency was 0.863, and the mean scale efficiency was 0.822. The analysis results indicate that, following the substitution of output indicators, there were no significant changes in the efficiency values, rankings, or scale of returns status for some departments. Consequently, the efficiency measurement results are to some extent sensitive to the treatment of output indicators; however, the results of the sensitivity analysis are intended solely to assist in assessing the robustness of the research findings and should not be interpreted as a complete validation of the conclusions drawn from the main model. Refer to Table 3.

**Table 3:** Analysis of clinical department operational efficiency based on a sensitivity model.

Department category	Department name	Pure Technical Efficiency	Scale efficiency	Comprehensive technical efficiency	Effectiveness	Returns to scale
Internal medicine	Cardiology	1	0.663	0.663	Weakly effective	drs
	Respiratory and critical care medicine	0.882	0.785	0.692	Inefficient	drs
	Integrative medicine	0.695	0.949	0.659	Inefficient	irs
	Gastroenterology	0.942	0.954	0.898	Inefficient	drs
	Neurology	0.542	0.892	0.483	Inefficient	drs
	Paediatrics	1	0.671	0.671	Weakly effective	drs
	Haematology	0.759	0.965	0.732	Inefficient	irs
	Oncology	1	1	1	Efficient	—
	Nephrology	0.61	0.936	0.571	Inefficient	irs
	Endocrinology	1	1	1	Efficient	—
	Infectious diseases	0.701	0.999	0.7	Inefficient	drs
	General practice	1	1	1	Efficient	—
	Rehabilitation medicine	0.763	0.776	0.592	Inefficient	irs
Surgery	Obstetrics and gynaecology	1	0.62	0.62	Weakly effective	drs
	Otolaryngology–head and neck surgery	1	1	1	Efficient	—
	Ophthalmology	1	1	1	Efficient	—
	Oral and maxillofacial surgery	1	1	1	Efficient	—
	Cardiothoracic surgery	1	1	1	Efficient	—
	Department of plastic surgery, wounds and burns	1	0.606	0.606	Weakly effective	irs
	Orthopaedics	0.836	0.594	0.496	Inefficient	drs
	General surgery	1	0.842	0.842	Weakly effective	drs
	Neurosurgery	1	0.699	0.699	Weakly effective	drs
	Urology	1	0.944	0.944	Weakly effective	irs
Total	Average	0.901	0.865	0.777		

#### 3.2.3 Analysis of slack in inefficient clinical departments

Analysis of slack variables revealed that some departments not classified as DEA-efficient exhibited redundancy in bed and physician inputs, while others showed insufficient output in terms of outpatient service volume or inpatient turnover. From the input perspective, the five departments with the highest bed redundancy rates were General Practice and Infectious Diseases, among others, with General Practice recording the highest rate at 0.391. Redundancy in physician input was particularly evident in departments such as the Department of Integrated Chinese and Western Medicine and the Department of Orthopaedics. From the output perspective, insufficient outpatient visits were concentrated in five departments, including neurosurgery and hematology; among these, neurosurgery had the highest rate of insufficient outpatient visits, reaching 17.312. Insufficient outputs related to inpatient efficiency were primarily concentrated in departments such as Orthopaedics and Neurosurgery; among these, Orthopaedics had the highest rate of insufficient inpatient output, at 2.110. Refer to Table 4.

**Table 4:** Slack variable analysis of inefficient clinical departments.

Department category	Department name	Input redundancy ratio			Output shortfall rate		
		Number of beds	Number of nurses	Number of doctors	Number of outpatient visits	Inverse of average length of stay	CMI-adjusted number of discharges
Internal medicine	Respiratory and critical care medicine	0	0	0.019	0.585	0.663	0
	Integrative Medicine	0	0	0.224	0	0.03	0
	Gastroenterology	0	0	0.096	0	0	0
	Neurology	0	0	0.049	0.181	0.535	0
	Haematology	0.156	0	0	3.405	0	0
	Nephrology	0.056	0	0.079	0	0.028	0
	Infectious diseases	0.334	0	0	0	0.173	0
	General practice	0.391	0	0.279	0	0	0
	Rehabilitation medicine	0.149	0	0.306	0	0.393	0
Surgery	Orthopaedics	0.067	0	0.138	0	2.11	0
	Neurosurgery	0.054	0	0	17.312	2.061	0
	Urology	0.218	0	0	0.437	0	0

### 3.3 Analysis of factors affecting operational efficiency in hospital clinical departments

To further analyze the statistical relationship between departmental contextual variables and the comprehensive technical efficiency of clinical departments, this study used the comprehensive technical efficiency values calculated via DEA as the dependent variable and incorporated departmental attributes, designation as a national or provincial key specialty, and the proportion of physicians with senior professional titles into the second-stage analysis. Refer to Table 5.

**Table 5:** Tobit regression analysis of factors associated with clinical department operational efficiency.

Variable	Regression coefficient	Standard error	*z*	*p*	95% confidence interval
Department attributes	0.033	0.074	0.438	0.661	−0.113 ~ 0.178
National or provincial key specialities	−0.129	0.075	−1.722	0.085	−0.275 ~ 0.018
Proportion of doctors with senior professional titles	0.645	0.211	3.056	0.002	0.232 ~ 1.059

*Department type and status as a national or provincial key specialty are categorical variables, with the following specific coding: Internal Medicine is coded as 0, and Surgery as 1; non-national or non-provincial key specialties are coded as 0, while national or provincial key specialties are coded as 1. Proportion of doctors with senior professional titles = number of doctors with senior professional titles/total number of doctors.

The Tobit model results indicate that the proportion of physicians with senior professional titles is significantly positively correlated with the comprehensive technical efficiency of clinical departments (*β* = 0.645, *p* = 0.002), suggesting that departments with a higher proportion of physicians holding senior professional titles exhibit relatively higher levels of comprehensive technical efficiency. The statistical associations between department attributes (*β* = 0.033, *p* = 0.661) and national or provincial key specialties (*β* = −0.129, *p* = 0.085) and overall technical efficiency did not reach statistical significance (*p* > 0.05).

## 4 Discussion

### 4.1 Efficiency analysis based on the DEA model

There are certain disparities between the medical and surgical departments within the sample hospitals in terms of resource allocation and operational efficiency; the average overall technical efficiency of the surgical departments is generally higher than that of the medical departments. Among the 13 medical departments, only the oncology and endocrinology departments achieved DEA efficiency. While some departments exhibited high pure technical efficiency, they lacked scale efficiency, suggesting that the primary issue may lie in a mismatch between the scale of bed and staff resources allocated and the actual service output. Within the surgical system, some departments have scale efficiencies close to or at 1; however, departments such as orthopaedics and neurosurgery still exhibit some losses in technical or scale efficiency. The DEA results indicate that the hospital should implement differentiated improvements for different types of inefficient departments, taking into account departmental functional positioning, case mix, and service capacity, rather than making uniform adjustments based solely on whether a department is classified as medical or surgical.

### 4.2 Analysis of correlated factors based on the tobit model

The results of the Tobit regression analysis indicated that the proportion of senior-level physicians was significantly and positively correlated with the comprehensive technical efficiency of clinical departments, while the statistical associations between departmental attributes and national or provincial key specialities and comprehensive technical efficiency did not reach statistical significance. The positive correlation between the proportion of senior-level physicians and overall efficiency may be explained by the fact that departments with a higher proportion of senior-level physicians possess certain advantages in terms of their ability to manage complex cases and the efficiency of clinical decision-making; however, this mechanism requires verification in subsequent studies incorporating clinical process and quality indicators. As this study is a cross-sectional analysis at the single-hospital, departmental level, and as DEA-Tobit analysis merely reflects statistical associations between variables, it is not possible to infer from the results that the proportion of senior-level physicians has a causal effect on operational efficiency. Improvements in the operational efficiency of clinical departments may also be related to staff structure, post-allocation, technical capabilities, case mix, and the degree of alignment between resource inputs and operational requirements.

### 4.3 Analysis of dual-model results

This study integrates the results of DEA, slack variable, and Tobit regression analyses to examine the operational efficiency characteristics of clinical departments from the perspectives of efficiency measurement, the identification of input–output slack, and the analysis of related factors. For departments identified as efficient by DEA, their resource allocation and operational characteristics can be further analyzed, and indicators such as healthcare quality and patient outcomes can be used to determine whether there are management practices that other departments could emulate; for departments identified as weakly efficient by DEA, the focus should be on issues of scale efficiency, assessing whether the scale of beds, staffing, and service output are appropriately matched; for departments identified as inefficient by DEA, adjustments should be made in conjunction with the results of the slack variables, with departments exhibiting input redundancy prioritizing the optimization of resource allocation; departments with insufficient output should prioritize improving service processes to enhance service capacity and resource turnover efficiency. It should be noted that a DEA efficiency score of 1 merely indicates that the department lies on the relative efficiency frontier within the scope of this study’s sample and the established input–output indicator system; it does not imply that its clinical quality, patient outcomes, cost-effectiveness, or absolute operational performance are superior to those of other departments (12).

## 5 Recommendations

### 5.1 Optimising the structure of bed and human resource allocation

Conduct an input–output evaluation of clinical departments that are not DEA-efficient, identify the causes of imbalances in resource allocation, and implement categorized remedial measures. Firstly, for departments that are inefficient but possess development potential, enhance service capacity and efficiency through technological upgrades, equipment renewal, or the recruitment of talent (9). Secondly, for departments with persistently low efficiency and limited scope for expansion, a hospital-wide, unified bed management mechanism should be established, and interdepartmental resource coordination mechanisms should be refined to promote dynamic bed turnover and centralized allocation. Existing research indicates that a refined, unified bed management model is conducive to improving bed utilization efficiency and optimizing the allocation of medical resources (13). Consequently, hospitals need to establish an efficiency-oriented strategy for managing available beds, thereby providing institutional safeguards for the optimization of resource allocation.

### 5.2 Strengthening the development of specialist capabilities

The development objective of “comprehensive care with specialized excellence” has been established. Through a differentiated operational approach of ‘one policy per department,’ departmental resources are rationally allocated to guide departments in exploring pathways to sustainable development (14). At the “comprehensive care” level, sustainable development plans are formulated for high-performing departments to ensure their efficient and stable operation. Measures such as resource coordination and process optimization enhance efficiency for departments that are small in scale or technologically inefficient. At the “strong specialist care” level, we utilize technological innovation to enhance the efficiency of resource utilization per unit. Resource allocation: Integrating the DRG performance evaluation system with the cost accounting system optimizes resource allocation and enhances the departments’ comprehensive treatment capabilities (15). We dynamically assess the number, structure, and professional background of medical and nursing staff to formulate differentiated talent development and recruitment plans. We dynamically assess the number, structure, and professional background of medical and nursing staff to formulate differentiated talent development and recruitment plans (16). By focusing on areas of clinical strength and key diagnostic and treatment fields, departmental collaboration and the development of multidisciplinary treatment models are promoted, thereby enhancing specialist diagnostic and treatment capabilities as well as service quality.

### 5.3 Establishing an efficiency-oriented departmental performance evaluation mechanism

Hospitals may utilize the results of DEA efficiency analysis, slack variables, and Tobit analysis as supplementary information for departmental operational management, thereby establishing a routine and dynamic mechanism for evaluating the operational efficiency of clinical departments. By measuring departmental efficiency through DEA models and utilizing slack variable analysis to identify resource redundancies and output shortfalls, hospitals can establish a data-driven, precise quality control system and quantifiable evaluation criteria. The results of these assessments can then serve as the basis for management decisions, enabling the identification of potential risks and opportunities for optimization in resource allocation (17). For departments with insufficient output, clear incremental targets and improvement pathways Departments with insufficient output define clear incremental targets and improvement pathways. Relying on information technology and intelligent management tools, a departmental operational monitoring mechanism encompassing early warning, analysis, and improvement is established. A comprehensive evaluation system incorporating inputs, outputs, quality, and efficiency is developed, with factors such as clinical quality and service satisfaction included in the evaluation indicators to avoid the problem of pursuing efficiency at the expense of quality.

## 6 Summary

Based on analyses using the DEA-BCC and Tobit regression models, there are significant differences in operational efficiency among the clinical departments of the sample hospitals, with inefficient departments primarily characterized by a mismatch between inputs and outputs. Hospitals should implement categorized management based on the functional roles and characteristics of economies of scale of each department, optimize the allocation of beds and human resources, and enhance their capacity for the diagnosis and treatment of complex and critical cases, as well as the efficiency of converting service inputs into outputs. The sample size in this study is limited, and the DEA efficiency scores may be influenced by the selection of input and output indicators, the comparability between departments, and outliers; the Tobit model incorporated a limited number of contextual variables, and cross-sectional data can only reflect statistical associations between variables, not infer causal relationships. Therefore, future research should expand the sample scope, conduct multi-year or multi-hospital panel studies, and further incorporate indicators such as case mix, diagnostic and therapeutic techniques, healthcare quality, patient outcomes, and cost-effectiveness to enhance the robustness and interpretability of the research conclusions.

## Data Availability

The original contributions presented in the study are included in the article/supplementary material, further inquiries can be directed to the corresponding author.
